# Peri-cardiac surgery coagulation management in a severe hemophilia A patient: A case report

**DOI:** 10.1097/MD.0000000000015897

**Published:** 2019-06-14

**Authors:** Hongfei Xu, Davies Henry, Chengcheng Li, Haige Zhao, Yanyan Yang

**Affiliations:** a Department of Cardiothoracic Surgery; b Department of pharmacy, The First Affiliated Hospital, Zhejiang University School of Medicine, Hangzhou, China.

**Keywords:** cardiac surgery, coagulation management, hemophilia A

## Abstract

**Rationale:**

Hemophilia A (HA) is an X-linked recessive disorder caused by clotting factor VIII (FVIII) deficiency. There is limited data on the use of replacement therapy in cardiac surgery. Since no international guideline for anticoagulation in such patient exists, careful thought should be taken to design an individualized anticoagulation strategy.

**Patient concerns:**

We report a 54-year-old male with severe HA with FVIII activity of 0.8% when he was first diagnosed, who underwent successful mitral valve repair and coronary artery bypass graft with FVIII replacement perioperatively.

**Diagnoses:**

Transthoracic echocardiography and coronary angiography confirmed the HA patient with the diagnosis of severe mitral valve regurgitation and left anterior descending artery stenosis.

**Interventions:**

Before surgery, a bolus of 1000 IU FVIII was injected, which obtained an FVIII of 80%. After induction, a 3750 IU bolus of FVIII was injected and subsequent FVIII level reached 135%. Mitral valve repair and coronary artery bypass graft with FVIII replacement were performed. After the surgery, a repeat FVIII activity level was 50.6%. The 400 mL of autologous blood and 700 mL of cardiopulmonary bypass (CPB) machine blood was returned to the patient as well as 4 units of fresh frozen plasma with an additional bolus of 1000 IU FVIII. 100 mg aspirin per day alone was given after surgery.

**Outcomes:**

The patient recovered uneventfully and 1-year follow-up showed no complications.

**Lessons:**

The anticoagulant or antiplatelet regimen of HA patient following surgery should be individualized based on the evaluation of the risk factors for bleeding and thrombosis and the lowest FVIII activity ever recorded after FVIII replacement therapy.

## 1 Introduction

Hemophilia A (HA) is an X-linked recessive disorder caused by clotting factor VIII (FVIII) deficiency. HA is classified according to plasma procoagulant levels into mild (5%–40%), moderate (1%–5%) and severe (<1%). There is limited data on the use of replacement therapy in those requiring cardiopulmonary bypass (CPB) for complicated cardiac surgery. Here we report a 54-year-old male with severe HA who underwent successful mitral valve repair (MVP) and coronary artery bypass graft (CABG) with FVIII replacement perioperatively. Since no international guideline for anticoagulation in such patient exists, careful thought should be taken to design an individualized anticoagulation strategy.

## 2 Case report

**Figure 1:**
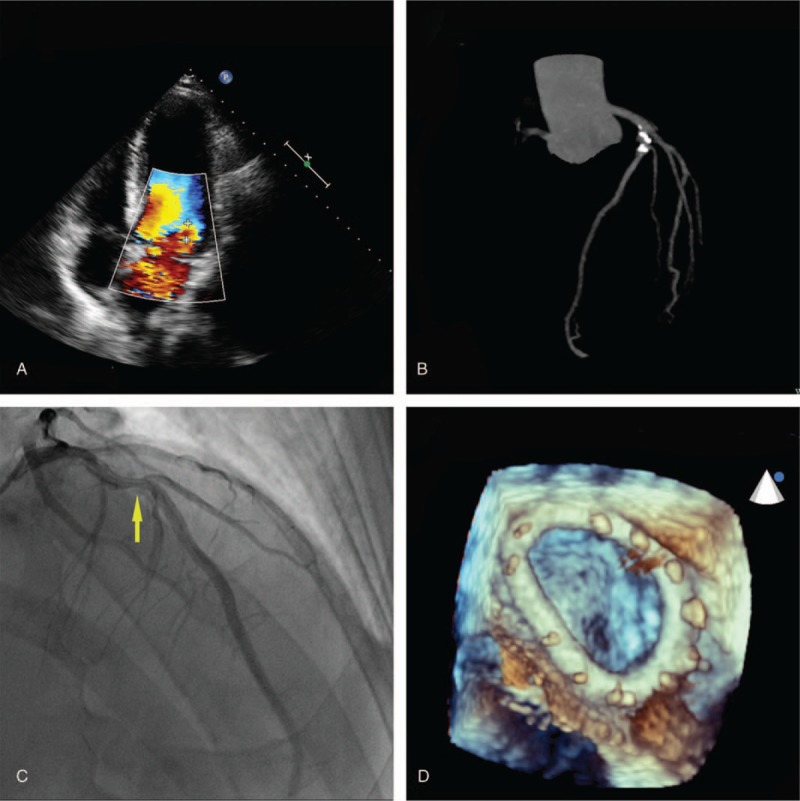
(A) Transthoracic echocardiographyshowed a posterior leaflet prolapse of mitral valve with moderate to severe regurgitation. (B) Coronary computed tomography angiography demonstrated calcification and moderate stenosis in the left anterior descending coronary artery (LAD). (C)Coronary angiography showed a 60% stenosis in the proximal LAD (yellow arrow). (D) Three-dimensional imaging of transesophageal echocardiography revealed no regurgitation after mitral valve repair with the use of an annuloplasty ring.

A 54-year-old male (body weight 75 Kg) was admitted to our hospital with acute chest tightness, asthma and shortness of breath after unexpectedly sliding down 3 steps 1 week ago. The patient had a history of HA with FVIII activity of 0.8% when he was first diagnosed. After the diagnosis, he received replacement therapy (13.3 IU/kg of FVIII each time) twice a week, which maintained an FVIII activity ranging from 2% to 80%. And he underwent bilateral knee replacement 5 years ago due to spontaneous hemorrhage. He has a 30-year history of smoking and drinking without withdrawal. Physical examination revealed a grade IV/VI holo-systolic murmur heard best at the apex and wet rales in both lungs. Transthoracic echocardiography (TTE) showed a posterior leaflet prolapse of mitral valve (P2 mainly) with moderate to severe regurgitation due to rupture of tendinae, and a left ventricular diastolic diameter of 56 mm and an ejection fraction of 70% (Fig. 1 A). Coronary computed tomography angiography (CTA) demonstrated calcification and moderate stenosis of the left anterior descending artery (LAD) (Fig. 1 B). Further examination of coronary angiography showed a 60% stenosis in the proximal LAD (Fig. 1C). Thus an operative plan of CABG and MVP was made.

The perioperative coagulation management strategy for this patient was based on the recommendations of anesthesiologist, surgeons, clinical pharmacists and perfusionists. Our goal was to achieve an initial FVIII level ≥70% for more than 2 weeks. Meanwhile, psychologists were counseled because patient was very anxious about excessive bleeding during surgery.

Two hours before surgery, a bolus of 1000 IU (13.3 IU/kg) of FVIII (Recombinant Coagulation FVIII for Injection, Bayer HealthCare LLC) was injected, which obtained an FVIII of 80%. After induction, a 3750 IU (50 IU/kg) bolus of FVIII was injected and subsequent FVIII level reached 135%. A tranexamic acid infusion of 1 g/h (lasting 10 h) was then started before incision. An intravenous introducer sheath (9F) was placed in the right internal jugular vein, and 400 mL of autologous blood was harvested. A dose of heparin (375 IU/kg) for CPB resulted in an activated clotting time (ACT) ≥300 seconds before the left internal mammary artery (LIMA) was severed. Surgery was performed with LIMA anastomosed to LAD after cardioplegic arrest, mitral valvuloplasty involving a triangular resection of the posterior leaflet, creation of an artificial chordae (Gortex CV-4) and insertion of an annuloplasty ring (28#, Sorin). The CPB lasted around 95 minutes, and a transesophageal echocardiography examination was performed (Fig. 1 D). Finally, heparin was neutralized with 220 mg of protamine, causing ACT to return to 130 seconds, and a repeat FVIII activity level was 50.6%. 400 mL of autologous blood and 700 ml of CPB machine blood was returned to the patient as well as 4 units of fresh frozen plasma with an additional bolus of 1000 IU (13.3 IU/kg) FVIII. The patient was transferred to the surgical intensive care unit and extubated within 2 hours postoperatively. 100 mg aspirin per day was given from that day on. A bolus of 2000 IU (26.7 IU/kg) FVIII was administered in the evening of the day of surgery, and FVIII supplementation was continued every 12 hours (2500 IU each time on days 1–4, 2000 IU on days 5–9 and 1000 IU on days 10–14) until postoperative day 14.

The total postoperative drainage from mediastinal, chest and pericardial drainage tubes was 350 mL on day 1 and 150 mL on day 2, so the tubes were removed on the 3rd postoperative day. The patient recovered uneventfully and was discharged home on the 8th postoperative day with a residual FVIII activity of 54%. One-year follow-up showed the patient had no bleeding or thrombosis complications, and TTE showed no evidence of mitral regurgitation.

## 3 Discussion

There are few reports of patients with HA undergoing cardiac surgery, only 7 cases of CABG combined with valve surgery have been reported in English literature,^[1]^ while there are currently no reports of patients with severe HA undergoing simultaneous CABG and MVP. HA patients’ FVIII activity can be returned to normal ranges through replacement therapy. Patrick odonkor^[2]^ summarized the perioperative coagulation management plan of HA patients, and successfully applied the strategy of FVIII replacement therapy on an actual case. When planning our coagulation strategy we referred to this paper, but made a slight adjustment, confirming that this is a viable strategy. Patients with HA will not benefit from atherosclerosis and the development of ischemic cardiovascular disease.^[3]^ Moreover, the number and quality of platelets in HA patients are normal. Therefore, our patient's LAD of 60% stenosis needed CABG surgery. Antiplatelet therapy is still the main pillar of secondary prevention after coronary artery surgery. It is recommended in American Heart Association guidelines (class I, level A) that aspirin should be used 81 to 325 mg/d before and 6 hours after CABG surgery, and should be used lifelong to reduce bridge obstruction and adverse cardiac events.^[4]^ Many patients with coronary artery disease are accompanied by valvular disease at the same time, so in recent years more and more patients receive simultaneous CABG and valve surgery. As for which antithrombotic therapy should be used after MVP, there is no clear consensus at the moment. The European guidelines recommend taking vitamin K antagonists for anticoagulation therapy (class IIA, level c) for 3 months post mitral valvuloplasty, but the application varies from country to country. American surgeons chose antiplatelet therapy (grade 2C), 64% of British surgeons chose warfarin for 6 months after MVP, while 54% of surgeons use aspirin long-term. Currently in China, most cardiac surgeons choose warfarin therapy for 3 months after MVP. Nevertheless, in the handful of HA patients underwent CABG plus valve surgery reports out there, a range of coagulation management plans were used, including heparin alone,^[5]^ warfarin alone,^[6]^ aspirin plus warfarin for 30 days,^[7]^ and not using anything at all.^[8]^ Aspirin is well tolerated in HA patients. Considering that our patient had severe HA and a history of bilateral knee replacement due to spontaneous bleeding with a low CHA2DS2-VASc score for chance of stroke and we only used a single LIMA bridge and an annuloplasty ring for MVP, his lowest FVIII activity after 1000 IU FVIII replacement therapy every other day was 8.1%, thus we came to the conclusion that 100 mg aspirin alone per day was suitable postoperatively. One year follow-up showed that the patient was well and had neither bleeding nor thrombosis event.

## 4 Conclusion

In conclusion, we believe that patients with severe HA can undergo simultaneous CABG and valve surgery if a suitable coagulation management strategy is enacted. Following surgery, the individual anticoagulant or antiplatelet regimen can be selected based on evaluation of the risk factors for bleeding and thrombosis and the lowest FVIII activity ever recorded after FVIII replacement therapy.

## Author contributions

**Conceptualization:** Yanyan Yang.

**Data curation:** Davies Henry.

**Investigation:** Chengcheng Li.

**Methodology:** Chengcheng Li.

**Validation:** Yanyan Yang.

**Writing – original draft:** Hongfei Xu.

**Writing – review & editing:** Haige Zhao.
